# Anpassung des Meldesystems gemäß Infektionsschutzgesetz im Jahr 2020 aufgrund von COVID-19

**DOI:** 10.1007/s00103-021-03298-w

**Published:** 2021-04-13

**Authors:** Michaela Diercke, Hermann Claus, Ute Rexroth, Osamah Hamouda

**Affiliations:** 1 ÖGD-Kontaktstelle: Fachgebiet Surveillance, Abteilung 3 – Infektionsepidemiologie, Robert Koch-Institut, Seestraße 10, 13353 Berlin, Deutschland; 2 Fachgebiet Infektionsepidemiologische Fach-IT und Anwendungsentwicklung, Robert Koch-Institut, Berlin, Deutschland; 3 ÖGD-Kontaktstelle: Fachgebiet für Infektionsepidemiologisches Krisenmanagement, Ausbruchsuntersuchungen und Trainingsprogramme, Robert Koch-Institut, Berlin, Deutschland; 4 Abteilung für Infektionsepidemiologie, Robert Koch-Institut, Berlin, Deutschland

**Keywords:** Öffentlicher Gesundheitsdienst (ÖGD), Surveillance, Infektionsschutzgesetz, Meldepflicht, Digitalisierung, Public health surveillance, Digitalization, Infectious diseases, COVID-19, Notification system

## Abstract

Die COVID-19-Pandemie hat Anpassungen des Meldesystems gemäß Infektionsschutzgesetz (IfSG) erforderlich gemacht. Da COVID-19 bis dahin unbekannt war, gab es noch keine spezifische Meldepflicht für diese Infektionskrankheit. Sie fiel jedoch unter die Meldepflicht für bedrohliche übertragbare Krankheiten nach § 6 Abs. 1 Nr. 5 IfSG. Sobald absehbar war, dass es sich nicht nur um Einzelfälle handeln würde, wurde die Meldepflicht zunächst per Verordnung angepasst und später in das IfSG aufgenommen. In diesem Beitrag werden die Anpassungen des Meldesystems beschrieben, die im Rahmen der COVID-19-Pandemie im Verlauf des Jahres 2020 erfolgten.

Neben der Einführung der Meldepflicht für COVID-19 wurden auch die Meldeinhalte erweitert, um Informationen, die speziell für COVID-19 relevant sind, erfassen zu können. Um den Arbeitsaufwand in Laboren und im Öffentlichen Gesundheitsdienst (ÖGD) beim Absetzen bzw. Verarbeiten der Meldungen zu reduzieren, wurde ein elektronisches Verfahren für Labormeldungen von SARS-CoV-2-Nachweisen eingeführt. Dies geschah im Rahmen der Entwicklung des Deutschen Elektronischen Melde- und Informationssystems für den Infektionsschutz (DEMIS). Zudem wurden umfangreiche Anpassungen in der Software für das Fall- und Kontaktpersonenmanagement vorgenommen.

Die an das Robert Koch-Institut (RKI) übermittelten Meldedaten bilden eine wichtige Grundlage für die Bewertung der epidemiologischen Situation und werden während der COVID-19-Pandemie tagesaktuell über diverse Wege zur Verfügung gestellt. Damit diese Daten immer zeitnah und in guter Qualität vorliegen, sollte die IT-Infrastruktur im ÖGD noch weiter modernisiert werden. Insbesondere sollte DEMIS wie geplant weiter ausgebaut werden.

## Abstract (en)

In response to the COVID-19 pandemic the routine surveillance system for infectious diseases had to be adapted. The disease was unknown before the first cases were reported under a catch-all notification requirement for new and threatening pathogens and diseases, but specific notification requirements for SARS-CoV‑2 detection by laboratories as well as for suspect cases of COVID-19 diagnosed by physicians were soon integrated in the infectious diseases protection act. This article describes how the notification system for infectious diseases was adapted in 2020 to meet the requirements of the COVID-19 pandemic.

In addition to the notification requirements, the list of data that is collected through the notification system was also amended. To facilitate the work of laboratories and local health authorities we have established the possibility for electronic reporting.

Additionally, the software used for case and contact management within the local health authorities had to be adapted accordingly.

COVID-19 notification data is important for the assessment of the current epidemiological situation and daily updated data was published by the Robert Koch Institute. To ensure timely data and good data quality, the IT infrastructure within the public health system has to be further modernized and the electronic notification system should be further strengthened.

## Einleitung

Die infektionsepidemiologische Surveillance ist ein essenzielles Instrument, um Infektionskrankheiten frühzeitig zu erkennen, deren Trend zu verfolgen und basierend auf den erhobenen Daten Infektionsschutzmaßnahmen zu implementieren und zu evaluieren. Besondere Herausforderungen entstehen beim Auftreten neuer Erreger und Krankheiten. In diesem Fall müssen Surveillance-Systeme flexibel angepasst werden, um auf die neuartigen Anforderungen zeitnah reagieren zu können.

In dieser Übersichtsarbeit werden Anpassungen des Meldesystems gemäß Infektionsschutzgesetz (IfSG) beschrieben, die im Rahmen der COVID-19-Pandemie im Verlauf des Jahres 2020 erfolgten, um aktuelle und zuverlässige Informationen für die Bewertung der epidemiologischen Lage bereitstellen zu können.

## Anpassung der Meldepflicht und Digitalisierung des Meldewegs

Damit die Gesundheitsämter frühzeitig Infektionsschutzmaßnahmen ergreifen können, müssen sie rechtzeitig über mögliche COVID-19-Fälle informiert werden. Dies geschieht im Rahmen des Meldesystems gemäß IfSG. Das IfSG legt fest, welche Infektionskrankheiten meldepflichtig sind, wer zur Meldung verpflichtet ist und welche Meldewege und -fristen eingehalten werden müssen, aber auch welche Inhalte die Meldungen enthalten dürfen und müssen.

Die Meldung muss unverzüglich erfolgen und dem Gesundheitsamt spätestens innerhalb von 24 h vorliegen. Die Meldung erfolgt namentlich. Im IfSG ist festgelegt, dass auch Name, Anschrift und Telefonnummer der betroffenen Person gemeldet werden müssen, damit das Gesundheitsamt sie frühzeitig kontaktieren, weitere Informationen zum Fall (z. B. klinische Informationen, Infektionsumfeld, Reiseanamnese) und Kontaktpersonen ermitteln und Infektionsschutzmaßnahmen anordnen kann.

### Meldungen von Laboren und anderen Meldepflichtigen an Gesundheitsämter

Um das Auftreten neuer Infektionskrankheiten im Meldesystem erfassen zu können, gibt es im IfSG sogenannte Auffangtatbestände. Gemäß § 6 Abs. 1 Nr. 5 IfSG sind „der Verdacht einer Erkrankung, die Erkrankung sowie der Tod, in Bezug auf eine bedrohliche übertragbare Krankheit“, meldepflichtig. Eine *bedrohliche* Krankheit ist dabei im IfSG als eine übertragbare Krankheit definiert, die aufgrund klinisch schwerer Verlaufsformen oder ihrer Ausbreitungsweise eine schwerwiegende Gefahr für die Allgemeinheit darstellen kann. Ebenso sind Labore gemäß § 7 Abs. 2 IfSG verpflichtet, Nachweise von im IfSG nicht explizit genannten Krankheitserregern zu melden, wenn unter Berücksichtigung der Art der Krankheitserreger und der Häufigkeit ihres Nachweises Hinweise auf eine schwerwiegende Gefahr für die Allgemeinheit bestehen. Auf dieser gesetzlichen Grundlage konnten auch schon vor dem Vorliegen der spezifischen Meldepflicht die ersten COVID-19-Fälle an die Gesundheitsämter gemeldet werden. Dies war essenziell für die frühzeitige Aufklärung des ersten Clusters, das in Bayern aufgetreten war [1].

Diese Auffangtatbestände unterliegen jedoch einem gewissen Interpretationsspielraum, sodass mit dem Auftreten weiterer Fälle in Deutschland zügig eine spezifische Meldepflicht für das Auftreten von COVID-19-Fällen eingeführt wurde. Gemäß § 15 IfSG können die Meldepflichten an die epidemische Lage angepasst werden. So kann das Bundesministerium für Gesundheit (BMG) in dringenden Fällen ohne Zustimmung des Bundesrates für den Zeitraum von 1 Jahr eine Rechtsverordnung erlassen, die die Meldepflicht ausdehnt. Auf Basis dieser gesetzlichen Grundlage ist zum 01.02.2020 die *Coronavirus-Meldepflichtverordnung* in Kraft getreten [2]. Mit dem *Zweiten Gesetz zum Schutz der Bevölkerung bei einer epidemischen Lage von nationaler Tragweite *wurde im Mai 2020 die Meldepflicht für den Verdacht einer Erkrankung, die Erkrankung und den Tod in Bezug auf COVID-19 sowie für den Nachweis von SARS-CoV‑2 in das IfSG integriert. Ebenso wurden auch die Meldeinhalte angepasst und erweitert, damit spezifisch für COVID-19 notwendige Angaben (z. B. das Infektionsumfeld) im Meldesystem erfasst werden können [3].

### Beschleunigte Einführung der elektronischen Labormeldung

Bereits vor der COVID-19-Pandemie wurden während größerer Ausbruchsgeschehen in Deutschland und auch in der täglichen Routine Schwächen des Meldesystems deutlich [4]. Meldungen erfolgen nicht medienbruchfrei und ressourcenschonend auf elektronischem Weg, steigende Anforderungen des Datenschutzes und der Datensicherheit können nicht vollumfänglich umgesetzt werden. Daher wurde bereits 2015 vom BMG ein Projekt zur Digitalisierung des Meldesystems initialisiert, in dessen Rahmen das *Deutsche Elektronische Melde- und Informationssystem für den Infektionsschutz (DEMIS)* entwickelt wird. Die gesetzliche Grundlage wurde mit dem *Gesetz zur Modernisierung der epidemiologischen Überwachung übertragbarer Krankheiten* im Jahr 2017 geschaffen [5].

Die wichtigsten Ziele von DEMIS sind:

- Umsetzung der elektronischen Meldung für alle Meldepflichtigen
- Digitale Unterstützung der Arbeit und Prozesse sowie des Datenmanagements in den Gesundheitsämtern
- Verbesserung der Kommunikation im Öffentlichen Gesundheitsdienst (ÖGD) durch zielgruppengerechte Veröffentlichung der Daten

Mit dem Anstieg der Fallzahlen in der ersten Welle der COVID-19-Pandemie wurde sehr schnell deutlich, dass die beschleunigte Umsetzung der elektronischen Labormeldung von SARS-CoV-2-Nachweisen wichtige Entlastungen bei Laboren und im ÖGD schaffen kann [6].

Entsprechend konnten das Robert Koch-Institut (RKI) und BMG in Kooperation mit dem Fraunhofer-Institut für offene Kommunikationssysteme (Fraunhofer FOKUS) und der gematik GmbH (Nationale Agentur für digitale Medizin) die erste Ausbaustufe von DEMIS (DEMIS-SARS-CoV-2) dank der bereits bestehenden umfangreichen Vorarbeiten innerhalb weniger Wochen zum Juni 2020 umsetzen.

**Figure 1:**
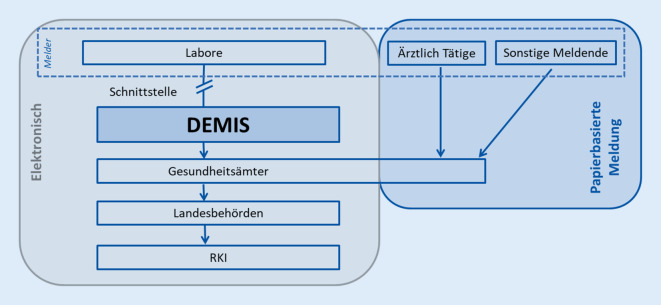

In der ersten Ausbaustufe von DEMIS wurde die elektronische Labormeldung von SARS-CoV-2-Erregernachweisen an die Gesundheitsämter über die zentrale DEMIS-Infrastruktur ermöglicht (Abb. 1). Damit wurde die Meldung per Fax und auf anderen Wegen abgelöst und die Gesundheitsämter müssen die Daten nicht mehr händisch in die Meldesoftware eingeben. Notwendige Ermittlungen und die anschließende Fallbearbeitung können somit unmittelbar durchgeführt werden.

Die Labore melden die Daten gemäß IfSG an die zentrale DEMIS-Infrastruktur. In dieser wird automatisiert bestimmt, welches Gesundheitsamt die Meldung empfangen darf, und die personenbezogenen Daten der Meldung werden für das zuständige Gesundheitsamt verschlüsselt. Die Gesundheitsämter können die Meldungen aus der zentralen DEMIS-Komponente elektronisch abrufen und die Daten direkt in ihre Software einlesen. Die Übermittlung der Fälle erfolgt weiterhin in der etablierten Infrastruktur und soll in einer späteren Ausbaustufe in DEMIS integriert werden.

Die elektronische Schnittstelle für die Labormeldung von SARS-CoV-2-Erregernachweisen wurde auf Basis des international etablierten FHIR-Standards (Fast Healthcare Interoperability Resources) umgesetzt.^1^ FHIR unterstützt den Datenaustausch zwischen Softwaresystemen im Gesundheitswesen und gewährleistet die notwendige Interoperabilität und damit die medienbruchfreie Informationsweitergabe zwischen den involvierten Akteuren.

**Figure 2:**
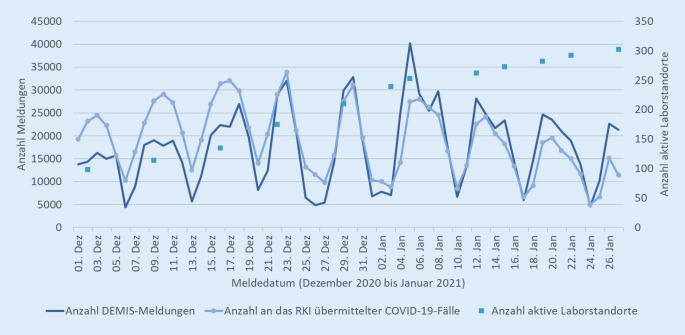

Mit dem *Dritten Gesetz zum Schutz der Bevölkerung bei einer epidemischen Lage von nationaler Tragweite* wurde für Gesundheitsämter und Labore zum 01.01.2021 die verpflichtende Nutzung von DEMIS für die Meldung von SARS-CoV-2-Nachweisen eingeführt [7]. Alle Gesundheitsämter konnten in der Roll-out-Phase seit Juni 2020 erfolgreich an DEMIS angebunden werden. Im Januar 2021 nutzten bereits über 300 Labore DEMIS aktiv zum Absetzen der Meldungen (Abb. 2).

Weitere Ausbaustufen von DEMIS sollen noch im Jahr 2021 umgesetzt werden. So soll für Arztpraxen, in denen Antigennachweise durchführt werden und die somit auch der Meldepflicht für den Nachweis von SARS-CoV‑2 unterliegen, ein Meldeportal zur Verfügung gestellt werden. Ebenso ist geplant, Funktionen für das automatisierte Zusammenführen von Nachmeldungen und Korrekturmeldungen zu implementieren und die Datenweitergabe zwischen den Gesundheitsämtern zu unterstützen. Zum 01.01.2022 sollen dann auch alle anderen Erregernachweismeldungen elektronisch an die Gesundheitsämter erfolgen. Die weitere Entwicklung von DEMIS soll durch den Pakt für den ÖGD zusätzlich unterstützt werden.^2^

## Digitale Unterstützung der Gesundheitsämter beim Fall- und Kontaktpersonenmanagement

Die beim Gesundheitsamt eingehenden Meldungen werden dort validiert und zu Fällen zusammengeführt. Das RKI stellt mit SurvNet@RKI^3^ eine kostenlose Software für die Gesundheitsämter zur Verfügung, mit der Daten zu gemeldeten Verdachts‑, Erkrankungs- und Todesfällen sowie Erregernachweisen seit 2001 elektronisch verarbeitet und an die zuständigen Landesbehörden und das RKI übermittelt werden können [8]. Am RKI und in den 16 zuständigen Landesbehörden wird SurvNet routinemäßig eingesetzt. Auch über 60 % der Gesundheitsämter nutzen SurvNet, was in diesen Ämtern Anpassungen, Aktualisierungen, aber auch Unterstützung durch das RKI erleichtert. Die übrigen Gesundheitsämter nutzen ein oder mehrere kommerziell verfügbare Softwareprodukte für diesen Bereich.

Während der Pandemie sind weitere Werkzeuge hinzugekommen, die die Gesundheitsämter z. B. beim Kontaktpersonenmanagement unterstützen sollen. In diesem Zusammenhang fördert das BMG z. B. den Einsatz der Software SORMAS^4^ in den Gesundheitsämtern, die im Rahmen eines Projekts über Schnittstellen auch an das Meldesystem angebunden werden soll, um Mehrarbeit in den Gesundheitsämtern zu vermeiden.

### Anpassung in der Gesundheitsamtssoftware

Nicht nur im Rahmen der Meldepflicht muss flexibel auf neu auftretende Infektionskrankheiten reagiert werden, sondern es muss auch die Möglichkeit bestehen, die Daten zu diesen Infektionskrankheiten elektronisch im Meldesystem zu erfassen. Die auf Grundlage der oben beschriebenen Auffangtatbestände gemeldeten Erkrankungen und Erregernachweise müssen im Gesundheitsamt elektronisch erfasst werden.

Auf Basis der Erfahrungen früherer Ausbruchsgeschehen und der Influenza-A(H1N1)-Pandemie im Jahr 2009 wurden folgende Prämissen für die Erfassung von Fällen und Kontaktpersonen während der COVID-19-Pandemie festgelegt:

1. *Integration ins bestehende Meldesystem*: Für die Erfassung von COVID-19-Fällen und -Kontaktpersonen sollte das bestehende Meldesystem gemäß Infektionsschutzgesetz genutzt werden. Die Fälle und Kontaktpersonen sollten in der derzeit im Gesundheitsamt verfügbaren Software erfasst werden. Soweit möglich, sollen keine zusätzlichen Datenerfassungen über separate Listen erfolgen. Dies soll sicherstellen, dass die Daten zu COVID-19 jederzeit verfügbar und standardisiert auswertbar sind. Durch die Integration in die im Gesundheitsamt vorhandene Software können vorhandene Werkzeuge (z. B. Abfragen) genutzt werden.
2. *Sichere Datenübermittlung*: Die Daten sollen sicher übermittelt werden. Dies kann über die Webservicefunktion in SurvNet@RKI sichergestellt werden. Zwischen Gesundheitsämtern, die den Webservice nutzen, zuständigen Landesbehörden und dem RKI können Daten sicher ausgetauscht werden.
3. *Strukturierte Erfassung der Daten*: Die Daten sollen in strukturierter Form und nicht als Freitext erfasst werden, damit die Datenerfassung standardisiert wird und Datenabfragen möglich sind. Analog zum Vorgehen bei der Meldung wurden die COVID-19-Fälle zunächst in der Auffangkategorie „Weitere bedrohliche Krankheiten“ in der Software erfasst. Es wurde jedoch ziemlich schnell deutlich, dass aufgrund des hohen Fallaufkommens, aber auch aufgrund von Zusatzinformationen, die erfasst werden mussten, eine eigene Übermittlungskategorie für COVID-19 in der Software geschaffen werden musste. Diese wurde im April 2020 mit der SurvNet-Version 0.9.26 eingeführt.

### Einheitliche Kriterien für die Bewertung der Meldedaten

Um die Gesundheitsämter bei der Bewertung der eingehenden Meldungen zu unterstützen, veröffentlicht das RKI sogenannte *Falldefinitionen*, in denen klinische, epidemiologische und labordiagnostische Kriterien festgelegt werden. Auf deren Basis kann die Entscheidung getroffen werden, ob ein Fall an das RKI übermittelt werden muss und welche der übermittelten Fälle in den Statistiken des RKI veröffentlicht werden.^5^

Die Falldefinitionen orientieren sich an den internationalen Standards der Weltgesundheitsorganisation (WHO) und des Europäischen Zentrums für die Prävention und die Kontrolle von Krankheiten (ECDC) und werden regelmäßig auf Basis der aktuellen epidemiologischen Erfordernisse und neuer wissenschaftlicher Erkenntnisse angepasst.

### Anpassung der Übermittlungsinhalte

Nicht nur Meldepflichten und Meldeinhalte wurden während der COVID-19-Pandemie angepasst, auch der Umfang und die Detailtiefe der Informationen, die pseudonymisiert an die zuständigen Landesbehörden und das RKI übermittelt werden dürfen, wurden erweitert. Dies ermöglicht zum Beispiel die bessere Erfassung des wahrscheinlichen Infektionsumfelds, eine kleinräumigere Bewertung des Infektionsgeschehens, molekulare Typisierungsergebnisse und erstmalig auch die Erfassung von im Gesundheitsamt angeordneten Maßnahmen.

### Unterstützung beim Kontaktpersonenmanagement

Schon zu Beginn der COVID-19-Pandemie wurde deutlich, dass das Kontaktpersonenmanagement ein entscheidender Baustein für die Eindämmung des Infektionsgeschehens ist. In diesem Zusammenhang wurden die Funktionen für das Kontaktpersonenmanagement in SurvNet@RKI deutlich erweitert. Neben der Erfassung von wichtigen Informationen zu Kontaktpersonen können mithilfe der Software Aufgaben organisiert, Anschreiben erstellt und Arbeitsprozesse dokumentiert werden.

## Bereitstellung von Informationen für die Öffentlichkeit und Entscheidungsträger

Das Interesse an den Meldedaten war nie so groß wie seit Beginn der COVID-19-Pandemie. Das RKI stellt seit Jahren wöchentlich aktualisierte Statistiken zu meldepflichtigen Infektionskrankheiten im Epidemiologischen Bulletin sowie online als interaktive Abfragemöglichkeit über SurvStat@RKI zur Verfügung. Mithilfe von SurvStat@RKI können die voraggregierten Meldedaten individuell abgefragt sowie Tabellen, Abbildungen und Karten erstellt werden.^6^ Einmal jährlich werden die Meldedaten im Infektionsepidemiologischen Jahrbuch ausführlich analysiert und auf Basis sorgfältiger epidemiologischer Bewertungen eingeordnet.^7^

Aufgrund des hohen Informationsbedürfnisses und der dynamischen Entwicklungen in der COVID-19-Pandemie wurde die Datenbereitstellung deutlich ausgeweitet. Mit dem COVID-19-Dashboard wird ein tagesaktueller, schneller und interaktiver Überblick über die COVID-19-Daten ermöglicht. Bis Mitte Januar 2021 wurde das mobile *COVID-19-Dashboard* für Smartphones und das desktopbasierte Dashboard jeweils über 110 Mio. Mal aufgerufen. Allein in Kalenderwoche 02/2021 waren es jeweils um die 600.000 Zugriffe pro Tag. Aktuelle Daten werden seit März 2020 täglich auf der Webseite des RKI, auf dem COVID-19-Dashboard, in SurvStat@RKI und im Lagebericht des RKI veröffentlicht.^8^ Zusätzlich werden Datentabellen mit aggregierten Daten zur Verfügung gestellt. Im Datenhub des Dashboards (Datenplattform mit Analysetools) können die Daten maschinenlesbar über eine Schnittstelle automatisiert abgerufen werden. Zahlreiche Anfragen seitens der Politik und der Medien werden auf Basis der zur Verfügung gestellten Daten beantwortet. Darüber hinaus kooperiert das RKI mit zahlreichen wissenschaftlichen Einrichtungen und stellt Daten zur Verfügung. Am RKI selbst werden die Daten regelmäßig in Bezug auf verschiedene Fragestellungen ausgewertet und die Ergebnisse in wissenschaftlichen Fachzeitschriften veröffentlicht [6, 9, 10]. Über TESSy (The European Surveillance System) werden mindestens wöchentlich Daten für das ECDC und die WHO zur Verfügung gestellt [11].

## Diskussion und Ausblick

Das bestehende Meldesystem gemäß IfSG ermöglichte während der COVID-19-Pandemie eine schnelle und zuverlässige Verfügbarkeit von belastbaren Daten zur Verbreitung von COVID-19 in Deutschland. Es konnte flexibel an die neuen Erfordernisse angepasst werden. Gleichzeitig werden viele Schwächen des Systems evident und dessen kontinuierliche Weiterentwicklung erwies sich als notwendig. Für keine andere Krankheit wurden bisher so viele Fälle (über 2 Mio. innerhalb eines Jahres) innerhalb des Meldesystems übermittelt. Nur aufgrund der bereits bestehenden und gut etablierten stabilen Infrastruktur und der leistungsfähigen Software (insbesondere SurvNet@RKI, das sowohl das Fall- und Kontatkpersonenmanagement in den Gesundheitsämtern als auch die Datenübermittlung zwischen Ländern und Bund sicherstellt) konnten diese großen Datenmengen bewältigt werden.

Aufgrund der Heterogenität der in den Gesundheitsämtern genutzten Softwareprodukte können dringend notwendige Updates der Software nur asynchron und mit Verzögerung ausgerollt werden. Wichtige Daten zu COVID-19-Fällen stehen daher nicht bundesweit einheitlich für die Bewertung der epidemiologischen Situation zur Verfügung. Die COVID-19-Pandemie und der Bedarf der Gesundheitsämter an digitaler Unterstützung hat sogar noch zu einer weiteren Diversifizierung und zur erhöhten Heterogenität der Softwarelandschaft geführt, da viele Gesundheitsämter zusätzliche digitale Werkzeuge zur Unterstützung der internen Abläufe nutzen bzw. selbst entwickelt haben [12]. Durch den Einsatz zusätzlicher Softwareprodukte werden zwar bestimmte Bedarfe in den Gesundheitsämtern abgedeckt, die Datenqualität im Meldesystem wird jedoch aufgrund fehlender Interoperabilität und unvollständiger oder fehlender Schnittstellen gefährdet. Dies führt zu Mehrarbeit, Zeitverzögerungen und sogar Datenverlusten.

Gleichzeitig konnte die Krise als Chance genutzt werden: Mit der zusätzlichen Unterstützung der Projektpartner wurde die erste Ausbaustufe von DEMIS erfolgreich implementiert. Auf dieser Grundlage sollte das System nun stetig weiterentwickelt und weitere Funktionen in DEMIS integriert werden, damit eine Homogenisierung der bestehenden IT-Strukturen im ÖGD erfolgen kann. So könnten größere Ausbruchsgeschehen, aber auch die tägliche Routine zukünftig besser bewältigt werden.

Der hohe Bedarf an aktuellsten Daten hat dazu geführt, dass die Veröffentlichung von Daten teilweise ohne ausreichende Qualitätssicherung erfolgt. Die spätere Aktualisierung von Daten und mögliche Artefakte, z. B. durch Schwankungen in der Inanspruchnahme der Gesundheitsversorgung und im Testverhalten, aber auch durch organisatorische Herausforderungen im ÖGD, erzeugen viel Unruhe in den Medien und in der Öffentlichkeit. Zwar kommt den tagesaktuellen Daten in der derzeitigen Berichterstattung eine hohe Aufmerksamkeit zu, dennoch ist aus epidemiologischer Sicht für die meisten Fragestellungen eine wochenaktuelle Berichterstattung sinnvoller. Diese ermöglicht eine bessere Bewertung von Trends und es kann in der Bewertung der epidemiologischen Situation auf qualitätsgesicherte Daten zurückgegriffen werden. Zudem werden dadurch auch die personellen Ressourcen geschont und unnötige Aufwände im ÖGD (einschließlich RKI) reduziert.

Nicht zuletzt sollte jedoch auch betont werden, dass die Meldedaten allein nicht ausreichend sind, um die epidemiologische Lage zu bewerten. Zum einen sollte sich das Meldesystem auf die Erfassung solcher Angaben beschränken, die für die Umsetzung des Infektionsschutzes bedeutend sind, auch um die Arbeitslast in den Gesundheitsämtern in einem angemessenen Rahmen zu halten. Nicht für alle Informationen ist eine Erfassung über das Meldesystem mit vertretbarem Aufwand möglich. Zudem ist nicht für alle Aspekte eine Vollerfassung nötig. Für die Bewertung der epidemiologischen Situation sind Daten, die im Rahmen von anderen Surveillance-Systemen, Datenerhebungen und Studien erhoben werden, unbedingt notwendig, nur so kann ein umfassendes Bild der Lage erzielt werden (siehe auch Beiträge von Goerlitz et al. und Seifried et al. in diesem Themenheft).

Zum anderen ist neben der infektionsepidemiologischen Lagedarstellung auch eine aktuelle, differenzierte und kleinräumige Übersicht über ergriffene Maßnahmen und verfügbare Ressourcen nötig (Personal- und Bettenkapazitäten, Impfdosen, Schutzausrüstung etc.), um die Lage umfänglich beurteilen zu können. Auch in diesen Bereichen wurden während der Pandemie Initiativen ergriffen, die aber noch weiterentwickelt werden müssen.

## Footnotes

1 Das Profil ist öffentlich unter folgendem Link verfügbar: https://simplifier.net/covid-19labormeldung.

2 Beschluss der 93. Gesundheitsministerkonferenz vom 04.09.2020: https://www.gmkonline.de/documents/gmk-beschluss_pakt-fuer-den-oegd_1599462671.pdf (zuletzt abgerufen am 28.01.2021).

3 SurvNet@RKI (RKI, Deutschland): https://survnet.rki.de/.

4 Surveillance Outbreak Response Management and Analysis System (HZI, Deutschland): https://www.sormas-oegd.de/.

5 Die aktuelle Falldefinition für COVID-19 ist unter folgendem Link verfügbar: https://www.rki.de/DE/Content/InfAZ/N/Neuartiges_Coronavirus/Falldefinition.pdf.

6 Individuelle Abfragen können erstellt werden unter: https://survstat.rki.de/.

7 Das Infektionsepidemiologische Jahrbuch ist verfügbar unter: www.rki.de/jahrbuch.

8 Die Daten sind verfügbar unter: https://corona.rki.de. Die Lageberichte werden auf der RKI-Webseite veröffentlicht: https://www.rki.de/DE/Content/InfAZ/N/Neuartiges_Coronavirus/Situationsberichte/Gesamt.html.
